# Metagenomic analysis of goat feces from Ogliastra (Sardinia, Italy)

**DOI:** 10.3389/frmbi.2024.1474497

**Published:** 2025-01-21

**Authors:** Monica Rosaria Molotzu, Piera Angela Cabras, Lisa Di Marcantonio, Rossano Atzeni, Nicolò Pietro Paolo Macciotta, Antonella Canu

**Affiliations:** ^1^ Struttura Complessa Controllo Microbiologico e Ispezione Alimenti, Istituto Zooprofilattico Sperimentale della Sardegna “G. Pegreffi”, Sassari, Italy; ^2^ Nuoro Complex Structure-Tortolì Territorial Center, Istituto Zooprofilattico Sperimentale della Sardegna “G. Pegreffi”, Tortolì, Italy; ^3^ Struttura Complessa Batteriologia e Sviluppo Antigeni Batterici, Istituto Zooprofilattico Sperimentale dell’Abruzzo e del Molise “G. Caporale”, Teramo, Italy; ^4^ Dipartimento Quantum e Calcolo ad Alte Prestazioni, Bioscienze e Studi Superiori, Centro di Ricerca CRS4 Sardegna, Pula, Cagliari, Italy; ^5^ Dipartimento di Agraria, Università degli Studi di Sassari, Sassari, Italy

**Keywords:** goats, CAE, metagenomic, galaxy, Ogliastra

## Abstract

With its constitutive and functional characteristics, the intestinal microbiota plays a crucial role in the health condition of the animals. Variations in the composition and gene expression of the intestinal microbiota are associated with the risk of the onset of various pathologies of the gastrointestinal tract and chronic inflammatory intestinal diseases. The objectives of this study were to evaluate the variability in the composition of the intestinal microbiota of goats of different breeds (Sarda, Maltese, and Alpine) farmed in different flocks of the region of Ogliastra (Sardegna, Italy) and to assess whether the type of feeding (natural pasture grazing-based versus intensive) could affect the intestinal bacterial composition. We also evaluated possible differences in the composition of the intestinal microbiota between healthy and Caprine arthritis encephalitis (CAE)-affected goats. The economic damage caused by this pathology is due to the reduction in milk production, with infected animals having greater susceptibility to contract diseases. The results of our study highlighted a statistically significant difference (*P* = 0.001–0.005) in the intestinal bacterial composition between the intensively managed flock and the other natural pasture-based flock.g In particular, a significantly greater abundance of *Acidoaminococcaceae* in the intensive flock was obgserved. Furthermore, a significantly greater abundance of *Prevotellaceae* was found in two localities in which, out of a total of 29 animals, only four tested negative for CAE. From these data, we deduced that the presence of *Prevotellaceae* can be an indication of the disease. This difference could be attributed to the farming system, the Cardedu farm being the only intensive one, and to the geographical distance of this location from the other sampling sites. Therefore, the results of the present study suggest that extensive or intensive farm management may affect the intestinal microbiota of goats.

## 1 Introduction

The microbiome describes a dynamic community of microorganisms that colonize organisms from birth onward. The microbiome can vary according to different factors such as host species, age, diet, health, reproductive status, and the external environment. Moreover, it is directly linked to the host’s health status, including metabolism, immunity, and development (Feng et al., 2018). The fecal microbiome is modified in response to transient changes in the host, but the abundance of some major groups of microorganisms is relatively stable throughout the life of the host. Thus, relative proportions of these groups may act as a signature of health and wellbeing, which is known as the host environment (Muegge et al., 2012). In particular, the relative ratio between the two dominant phyla in mammalian fecal microbiomes, *Firmicutes* and *Bacteroidetes*, can be used to distinguish between carnivorous and herbivorous mammals, as each group is responsible for different metabolic demands (Kreisinger et al., 2018).

Monitoring the composition of the fecal microbiome throughout the life of animals can help assess their health status (Bahrndorff et al., 2016). For example, many domestic mammal species suffer from poor health, at least partially related to dysbiosis of the fecal microbiome and to a reduced microbial diversity (McKenzie et al., 2017).

Research on this topic has evolved rapidly thanks to new technologies using next-generation sequencing platforms, which have allowed the study of communities of microorganisms (metagenomics) (Satam et al., 2023). The gastrointestinal tract is a complex system that includes a fecal content characterized by more than 10^12^ bacteria per gram of feces, which is named the “microbiota” (Randeni et al., 2024). The genome of the intestinal microbiota is at least 100 times greater than that of the entire individual, and it is defined as the “microbiome.” The term metagenomics refers to the application of modern gene sequencing techniques to the study of microbial communities directly in their natural environment, thus bypassing the need to isolate and cultivate them in the laboratory (Chaudhari et al., 2023; Nam et al., 2023). These techniques have allowed the reconstruction of a large number of metagenome-associated genomes (MAGs) in different animal organisms, including goats, cattle, pigs, sheep, rodents, and poultry, and the detection of associations with host health and some illnesses (Consiglio Superiore di Sanità – Sezione III, 2018).

The 16S ribosomal RNA is a sequence that is shared universally by all prokaryotes, and it has extremely conserved regions interspersed with highly variable regions V1–V9 characterized by variable length and degree of diversity (Bertolo et al., 2024; Hrovat et al., 2024). These can be amplified and sequenced thanks to the use of degenerate primers designed on their flanking regions. The sequencing of hypervariable regions of bacterial 16S rRNA allows for the so-called metataxonomy or *phylotyping* of the microbial community itself, with the identification and assignment of the relative distributions of the so-called “taxonomic operational units” (OTUs) at different phylogenetic levels and the estimation of their relative abundances (Willis et al., 2019).

Ruminants are herbivorous hoofed mammals with specialized anatomical and physiological adaptations that make them able to perform cellulolytic fermentation of plant materials with a high content of fiber fractions. The study of the fecal microbiome of ruminant species could provide useful tools for developing strategies aimed at improving the animal health status, enhancing the ability to adapt to environmental changes, and preventing disease and parasite epidemics (McKenzie et al., 2017). Caprine arthritis encephalitis (CAE) is an infectious disease first reported in 1974 caused by a virus from the retrovirus family (Crawford et al., 1980; Narayan et al., 1980). The economic consequences of CAE are manifold. Apart from the reduction of milk production, infected animals are more susceptible to several diseases. Such a higher vulnerability not only enhances the risk of secondary infections but also increases the need for veterinary interventions, leading to an increase in operational costs for farmers. Moreover, this affliction contributes to the reduction of the longevity of infected animals, diminishing their overall productive life and, therefore, further enhancing the negative economic impact. Addressing the multifaceted challenges posed by CAE-related viral encephalitis arthritis requires a comprehensive approach that considers both the immediate losses in milk production and the long-term consequences for the health and productivity of livestock (Peterhans et al., 2004; Le Jan et al., 2005).

The study focused on the metagenomic analysis of goat feces, mainly of the Sarda breed, from six locations considered representative of the Ogliastra region, an area of Sardinia where the breeding of the Sarda goat is widespread: Baunei, Cardedu, Perdasdefogu, Talana, Urzulei, and Villagrande.

The study was carried out in the municipalities of Ogliastra (central-eastern Sardinia), included in a blue zone (demographic and/or geographical areas of the world, identified by the scholar Prof. Gianni Pes in which a higher concentration of centenarians is recorded) (Pes and Pouland, 2014). Currently, purebred Sardinian goats are farmed mostly in marginal areas, and crossbreeding with selected breeds (e.g., Murciano-Granadina and Alpine) is a common practice for improving milk yield and slaughter weight of kids. This breed, present on the island since the Neolithic, is characterized by a relevant genetic heterogeneity due to selection performed by shepherds and crosses with other breeds. In particular, three subpopulations differing in size (large, medium, and small), somatic features, and production levels can be distinguished (Macciotta et al., 2002). The Sarda breed goat is well adapted to the harsh environment of some areas of Sardinia where, despite the very difficult farming conditions (Usai et al., 2004), it produces milk and meat of excellent quality.

The aim of the study was to evaluate the differences between the microbial communities present and how the intestinal bacterial composition could be differentiated according to geographical location, type of feeding management (pasture-based or intensive), and CAE status (positive or negative).

## 2 Materials and methods

### 2.1 Sampling

The study area was the Ogliastra, in central-eastern Sardinia (Italy), with an extension of 1,855 km^2^ and a population of approximately 58,000 inhabitants, distributed in 23 localities of particular naturalistic interest.

**Figure 1:**
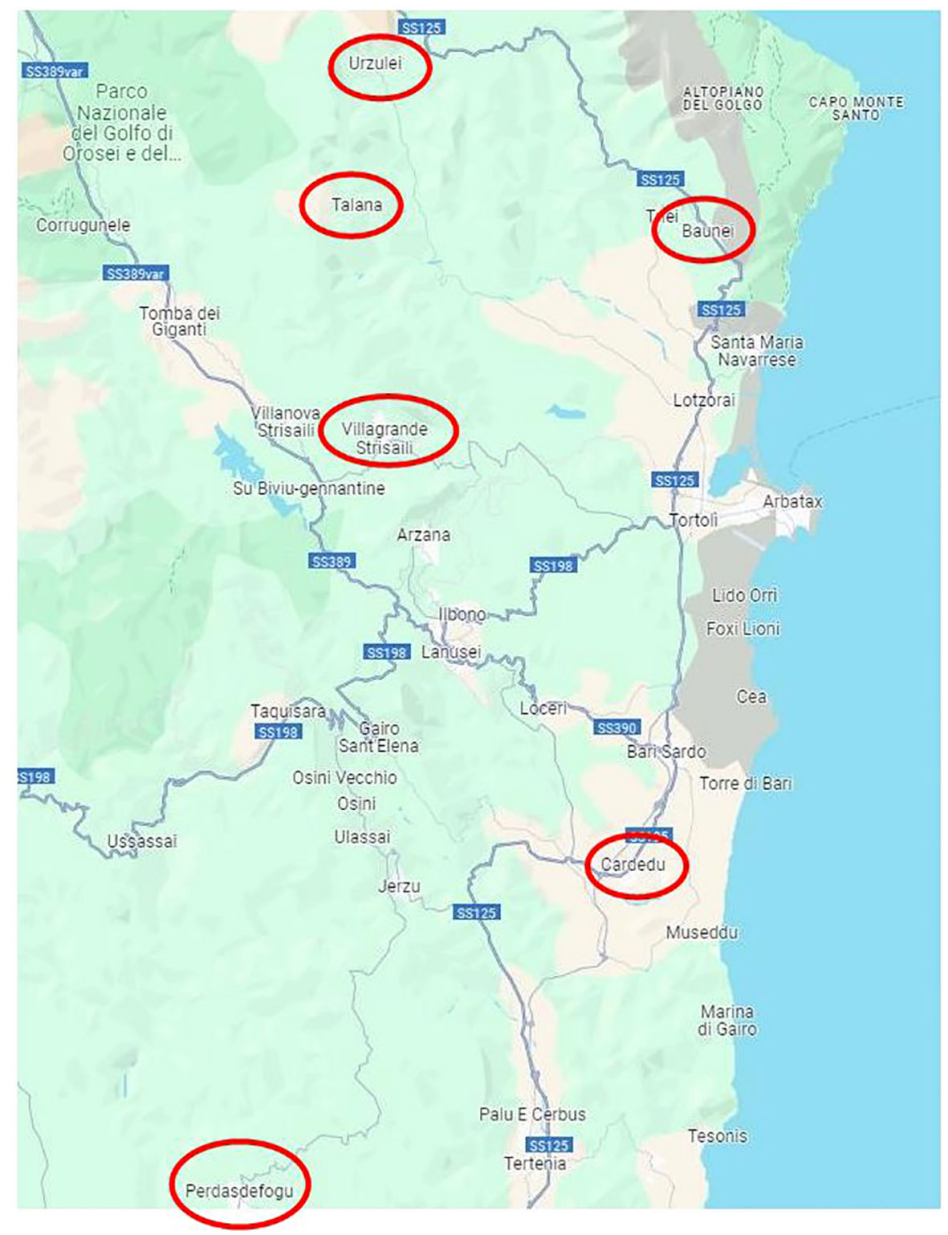
Sampling map.

Sampling was carried out between 07 August 2019 and 24 June 2021 on 19 goat flocks distributed in six different localities, representative of the goat farming system of the considered area. (Details on the animals analyzed for each farm and the sampling areas are described in **Table 1** and **Figure 1**).

**Table 1:** Sampling data.

Sampling data						
Sample	Sampling date	Locality	Sex	Age	Kind	CAE
1	29/03/2021	Baunei	Female	6	Sardinian	Negative
2	29/03/2021	Baunei	Female	3	Sardinian	Negative
3	29/03/2021	Baunei	Female	7	Sardinian	Negative
4	29/03/2021	Baunei	Female	3	Sardinian	Negative
6	29/03/2021	Baunei	Female	7	Sardinian	Positive
7	29/03/2021	Baunei	Male	3	Sardinian	Positive
8	18/03/2021	Baunei	Female	6	Sardinian	Negative
9	18/03/2021	Baunei	Female	5	Sardinian	Negative
10	18/03/2021	Baunei	Female	2	Sardinian	Positive
11	18/03/2021	Baunei	Female	4	Sardinian	Negative
12	18/03/2021	Baunei	Female	5	Sardinian	Positive
13	05/03/2021	Baunei	Female	5	Sardinian	Negative
14	05/03/2021	Baunei	Female	3	Sardinian	Negative
15	05/03/2021	Baunei	Female	6	Sardinian	Negative
16	05/03/2021	Baunei	Female	4	Sardinian	Negative
17	05/03/2021	Baunei	Female	3	Sardinian	Negative
19	05/03/2021	Baunei	Female	13	Sardinian	Positive
20	05/03/2021	Baunei	Female	12	Sardinian	Positive
21	05/03/2021	Baunei	Female	8	Sardinian	Positive
22	05/03/2021	Baunei	Female	3	Sardinian	Positive
24	16/03/2021	Talana	Female	3	Sardinian	Positive
25	16/03/2021	Talana	Female	4	Sardinian	Positive
26	16/03/2021	Talana	Female	3	Sardinian	Positive
27	16/03/2021	Talana	Female	6	Sardinian	Positive
28	16/03/2021	Talana	Female	5	Sardinian	Positive
29	16/03/2021	Talana	Female	6	Sardinian	Positive
30	16/03/2021	Talana	Female	6	Sardinian	Positive
31	16/03/2021	Talana	Female	5	Sardinian	Positive
32	16/03/2021	Talana	Female	6	Sardinian	Positive
33	16/03/2021	Talana	Female	5	Sardinian	Negative
34	25/03/2021	Urzulei	Female	6	Sardinian	Positive
35	25/03/2021	Urzulei	Female	3	Sardinian	Positive
36	25/03/2021	Urzulei	Female	3	Sardinian	Positive
37	25/03/2021	Urzulei	Female	4	Sardinian	Positive
38	25/03/2021	Urzulei	Female	3	Sardinian	Positive
39	25/03/2021	Urzulei	Female	1	Sardinian	Positive
40	25/03/2021	Urzulei	Female	5	Sardinian	Positive
41	25/03/2021	Urzulei	Female	3	Sardinian	Positive
42	25/03/2021	Urzulei	Female	6	Sardinian	Positive
43	31/03/2021	Urzulei	Female	2	Sardinian	Positive
44	31/03/2021	Urzulei	Female	8	Sardinian	Positive
45	31/03/2021	Urzulei	Female	4	Sardinian	Positive
46	31/03/2021	Urzulei	Female	2	Sardinian	Positive
47	31/03/2021	Urzulei	Female	5	Sardinian	Negative
48	07/04/2021	Urzulei	Female	3	Sardinian	Positive
49	07/04/2021	Urzulei	Female	5	Sardinian	Positive
50	07/04/2021	Urzulei	Female	7	Sardinian	Positive
51	07/04/2021	Urzulei	Female	3	Sardinian	Positive
52	07/04/2021	Urzulei	Female	3	Sardinian	Negative
53	07/04/2021	Urzulei	Female	5	Sardinian-Samen	Positive
54	01/03/2021	Villagrande	Female	7	Sardinian	Positive
55	01/03/2021	Villagrande	Female	6	Sardinian	Negative
56	01/03/2021	Villagrande	Female	4	Sardinian	Positive
57	01/03/2021	Villagrande	Female	6	Sardinian	Positive
58	01/03/2021	Villagrande	Female	3	Sardinian	Negative
59	01/03/2021	Villagrande	Female	16	Sardinian	Positive
60	12/03/2021	Villagrande	Female	4	Sardinian	Negative
61	12/03/2021	Villagrande	Female	5	Sardinian	Positive
62	12/03/2021	Villagrande	Female	7	Sardinian	Positive
63	12/03/2021	Villagrande	Female	3	Sardinian	Positive
3	12/03/2021	Villagrande	Female	5	Sardinian	Positive
66	12/03/2021	Villagrande	Female	N.D	Sardinian	Positive
67	12/03/2021	Villagrande	Female	N.D	Sardinian	Positive
68	12/03/2021	Villagrande	Female	N.D	Sardinian	Positive
69	12/03/2021	Villagrande	Female	2	Sardinian	Positive
70	12/03/2021	Villagrande	Female	4	Sardinian	Positive
71	24/03/2021	Cardedu	Female	4	Alpine	Positive
72	24/03/2021	Cardedu	Female	5	Maltese-Suede	Positive
73	24/03/2021	Cardedu	Female	6	Maltese	Positive
74	24/03/2021	Cardedu	Female	2	Saanen-Suede	Positive
75	24/03/2021	Cardedu	Female	3	Maltese-Saanen	Positive
76	09/04/2021	Talana	Female	5	Sardinian	Positive
77	09/04/2021	Talana	Female	1	Sardinian	Negative
78	09/04/2021	Talana	Female	2	Sardinian	Positive
79	09/04/2021	Talana	Female	7	Sardinian	Positive
80	09/04/2021	Talana	Female	2	Sardinian	Negative
81	14/04/2021	Perdasdefogu	Female	4	Sardinian	Positive
82	14/04/2021	Perdasdefogu	Female	5	Sardinian	Positive
83	14/04/2021	Perdasdefogu	Female	3	Sardinian	Positive
84	14/04/2021	Perdasdefogu	Female	5	Sardinian	Positive
85	14/04/2021	Perdasdefogu	Female	4	Sardinian	Negative
86	14/04/2021	Perdasdefogu	Female	5	Sardinian	Positive
98	05/11/2019	Arzana	Female	3	Sardinian	Positive
99	24/06/2021	Baunei	Female	8	Sardinian cross	Negative
100	24/06/2021	Baunei	Female	3	Sardinian	Negative
101	24/06/2021	Baunei	Female	4	Sardinian	Negative
102	24/06/2021	Baunei	Female	8	Sardinian	Negative
103	24/06/2021	Baunei	Female	5	Sardinian	Negative

In most of the farms, the animals were fed natural pastures, with concentrate supplementation during the manual milking or when animals were kept in the barn. An exception was the farm located in Cardedu, characterized by intensive management, with mechanical milking and the use of antibiotics and pesticides. Natural pastures were characterized by a high presence of Mediterranean scrub. Most represented plant species were *Erica*, *Arbutus unedo*, *Pistacia lentiscus*, *Myrtus communis*, *Allium subhirsutum*, *Ferula communis*, *Phillies angustifolia*, *Genista Corsica*, *Calycotome villosa*, *Olea europea*, *Pyrus amygdaliformis*, *Quercus ilex*, *Quercus suber*, *Rosmarinus officinalis*, and *Thymus capitatus. Cistus* species are chemically characterized by a high content of cellulose and xylan (Duarte et al., 2013).

Individual blood samples were collected using two vacutainer tubes. Stool samples were taken from the rectal ampoule of each animal. The samples were transported to the lab at a controlled temperature of 4°C.

### 2.2 CAE detection

The detection of the CAE virus was performed in serum samples using indirect ELISA with specific anti-small ruminant lentivirus (SRLV) antibodies. SRLVs are a group of genetically and antigenically heterogeneous RNA viruses belonging to the *Retroviridae* family and the *Lentivirus* genus. The viruses responsible for ovine Maedi-visna (MVV) and CAE, respectively, are grouped under the denomination of SRLVs (WOAH, 2021).

### 2.3 Processing, extraction, and library preparation for metagenomics

Metagenomic analysis was performed on the feces of 87 dairy goats (**Supplementary Material 1**) frozen at −80°C after collection. After thawing, 300 mg of feces was weighed, and 800 µL of NucliSENS Lysis Buffer from bioMérieux (Florence, Italy) (Bartels et al., 2003; Loens et al., 2003; Van Deursen et al., 2003) was added. The tubes were incubated at 90°C for 10 min with shaking at 1,400 rpm. Samples were centrifuged at 12,000 rpm for 4 min, and 500 µL of supernatant was then taken and subjected to nucleic acid extraction following the instructions provided by the manufacturing company bioMerièux. The DNA concentration of the samples was measured by a “Qubit^®^ 3.0 Fluorometer” using the “High sensitivity assay kit Qubit^®^ dsDNA” kit (Invitrogen, Carlsbad, CA, USA) (Qubit™ 4 Fluorometer Catalog Number Q33226 Publication Number MAN0017209 Revision D.0, Invitrogen). We performed several experiments starting from 100 mg of feces and 500 µL of lysis buffer up to 300 mg of feces, 800 µL of lysis buffer, and 100 µL of silica. This preparation allowed us to reach 3 ng/µL, necessary for metagenomic sequencing as required by the library preparation manual.

A concentration of 3 ng/µL genomic DNA (gDNA) was used for the amplification of the specific DNA region of the extracted samples, using the ranges V 2-4-8 and V 3-6-7 as primer sets −9 of the hypervariable region of the ribosomal 16S. The preparation of the libraries was carried out using the instructions of the manufacturer Thermo Fisher Scientific (Waltham, Massachusetts, USA) and the following kits: Ion 16S Metagenomics Kit, Ion Plus Fragment Library Kit, Agencourt™ AMPure, 70% ethanol, and a high-sensitivity assay kit (Qubit^®^ dsDNA) (Ion 16S™ Metagenomics Kit Catalog Number A26216 Publication Number MAN0010799 Revision C.0, Thermofisher).

### 2.4 Metagenomic sequencing

Six chips of Ion Chip 318 v2 prepared with the Ion Chef™ Instrument were used. Sequencing was performed with the Ion Personal Genome Machine (PGM) (Ion Chef™ Instrument user guide Maintenance, calibration, and troubleshooting Catalog Number 4484177 Publication number MAN0018668 Revision A.0, Thermofisher; Ion Personal Genome Machine™ (PGM™) System reference guide Catalog Number 4462921, Publication number MAN0009783 Revision A.0, Thermofisher).

### 2.5 Bioinformatic and statistical analysis

The data analysis was performed using different platforms as described below.

Ion Reporter v. 5.20.2.0 was used in the data analysis. The QIIME2 software suite v2021.4.0 was used to analyze the amplicon data of the 16S rRNA gene. A “manifest file” was created using the “Fastq manifest” command to import the raw FASTQ data. The DADA2 pipeline was used to denoise the sequences and remove chimeric sequences. The Silva database (arb-silva.de) was used to BLAST search the obtained sequences and determine the phylogeny of the OTUs. The naive Bayes classifier trained on the SILVA 99% consensus taxonomy was employed to assign taxonomy into OTUs, which can be accessed at https://data.qiime2.org/2021.4/common/silva-138-99-nb-classifier.qza. To evaluate the completeness of the microbial communities, we conducted a rarefaction analysis using Faith’s PD, Shannon, and observed OTU indices. The alpha diversity indices (observed OTU and Chao1) were computed, and the beta diversity metrics with unweighted UniFrac distances were calculated (Pielou, 1966).

We utilized the Emperor tool to generate Emperor plots for unweighted UniFrac distance and explored the principal coordinate (PCoA) plots in the context of the sample metadata (Vázquez-Baeza et al., 2013).

To calculate the group significance between the alpha and beta diversity indices, we used the Kruskal–Wallis (pairwise) test for the beta-group significance command. Furthermore, the beta-group significance command in the diversity plugin was utilized to test the distances between samples within a group. Finally, the statistical analysis was conducted using PERMANOVA with 999 permutations (Anderson, 2001).

In our study, we employed Mothur version 1.39.5, which was integrated into the Galaxy version 22.05 platform (Schloss et al., 2009; Schloss, 2020; The Galaxy Community, 2022). Following Mothur’s recommended guidelines, we adopted the “Chappid” pipeline (Chappidi et al., 2019) to structure our metagenomic analysis with established best practices.

First, we organized FASTQ files by geographic origin in a Galaxy workspace. Quality control involved FastQC (version 0.11.9) (https://www.bioinformatics.babraham.ac.uk/projects/fastqc/), followed by TrimGalore (version 0.6.7) (https://github.com/FelixKrueger/TrimGalore.com/fenderglass/Flye) for removing low-quality reads and adapters (using default parameters with a quality threshold of 20; QPhred).

For multisample analysis, we created a group file in FASTA format using Mothur’s Make.group. Removal of duplicate sequences was done with Mothur’s unique.seqs. Abundance tables for taxonomic classification and OTUs were generated using count.seqs (Schloss, 2013).

The data quality was assessed using summary.seqs, revealing sequences predominantly within the 125 to 290 base range. Subsequent data cleaning steps included screen.seqs for the systematic removal of low-quality reads and sequences.

After aligning with the Silva database (Quast et al., 2012), we observed most sequences between positions 6,212 and 13,871. To ensure complete overlap, we employed screen.seqs, filter.seqs, unique.seqs, and pre.cluster.

Chimera identification was performed with chimera.vsearch, and removal utilized remove.seqs. Taxonomic assignments via classify.seqs utilized the RDP reference taxonomy (Cole et al., 2013). Lineages were removed using remove.lineage for specific groups.

Cluster.split at the order level, Make.shared, and classify.otu provided OTU information (Wooley et al., 2010).

Krona was visualized by converting the Mothur taxonomy to Krona format and using the Krona pie charts and plots per sample (Ondov et al., 2011).

Normalization involved counting sequences per sample and subsampling using subsamples. Alpha diversity estimation was performed using rarefaction curves generated by Rarefaction.single and visualized with Galaxy’s plotting tool. A comprehensive summary report was produced by Summary.single, including metrics such as observed richness, coverage, the inverse Simpson index, and the total number of sequences.

For beta diversity, the thetaYC and Jaccard indices were calculated using Dist.shared, with visualization through Heatmap.sim. Venn diagrams and dendrograms were generated using the Venn and Tree.shared tools (Cuccuru et al., 2014) (**Supplementary Material 1**).

## 3 Results

Six runs were carried out with Ion PGM, which generated 20,590,826 reads with an average of 225,670 reads per sample.

We used the V2-4-8 and V3-6-7 regions because they were included in the sequencing kit. Initially, we analyzed the V4 region alone, which is widely used in the literature, but found the data to be less informative compared to the combined analysis of all regions. The quality of the sequences, assessed using the summary.seqs command, confirmed that most reads fell within the range of 125–290 bases. This multiregion approach enhanced taxonomic resolution and community representativeness, justifying its use despite being less conventional.

**Figure 2:**
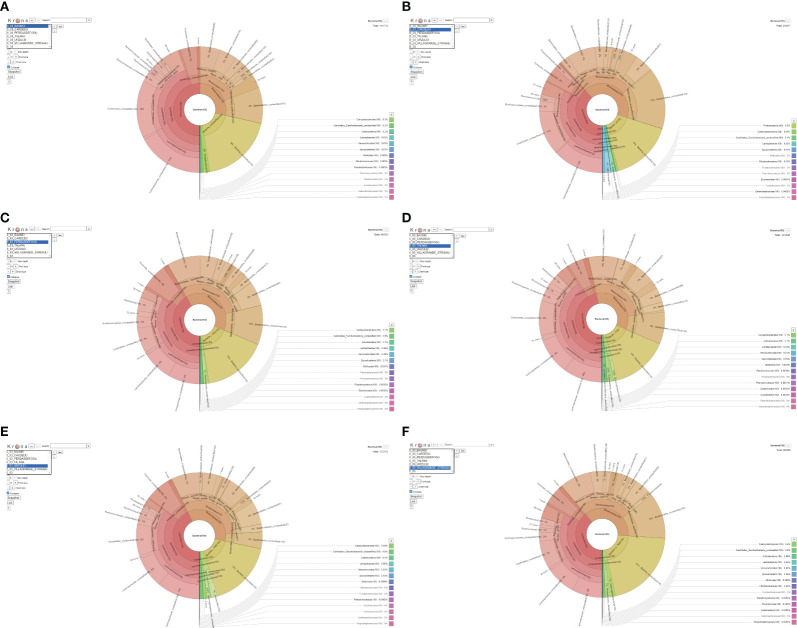
**(A-F)** Krona.

The microbiota of goat feces was mainly composed of the phyla *Firmicutes* and *Bacteroidetes*. In all the farms, except the one in Cardedu, an abundance of *Firmicutes* was noted compared to *Bacteroidetes*; in the farm in Baunei, the ratio was strongly unbalanced in favor of *Firmicutes*. In all the farms, there was a lower abundance of *Proteobacteria*, *Actinobacteria*, and *Verrucomicrobia*, except the farm in Cardedu, which has a slightly higher percentage than the others (**Figure 2**; **Table 2**).

**Table 2:** Phylum percentages obtained from the Krona graph.

Phylum percentages obtained from the Krona graph						
	Baunei	Cardedu	Perdasdefogu	Talana	Urzulei	Villagrande Strisaili
Firmicutes	50	36	42	47	43	39
Bacteroidetes	28	41	40	35	36	37
Proteobacteria	2	0.50	1	3	4	3
Actinobacteria	0.20	1	0.1	0.1	0.1	0.09
Verrucomicrobia	0.03	2	0.02	0.1	0.01	0.02

The most represented families were as follows: *Gracilibacteraceae*, *Erysipelotrichaceae*, *Clostridiales Family XI. Incertae Sedis*, *Christensenellaceae*, *Acidaminococcaceae*, *Peptostreptococcaceae*, *Clostridiaceae*, *Porphyromonadaceae*, *Prevotellaceae*, *Flavobacteriaceae*, *Eubacteriaceae*, *Ruminococcaceae*, *Lachnospiraceae*, *Rikenellaceae*, *Synergistaceae*, and *Bacteroidaceae*. The *Erysipelotrichaceae* family has a higher abundance in the Talana, Urzulei, and Perdasdefogu farms. The *Acidaminococcaceae* and *Porphyromonadaceae* families were very abundant in the Cardedu farm. The *Peptostreptococcaceae* family had a higher abundance in the Talana and Perdasdefogu farms, while *Peptostreptococcaceae* were present in very low percentages in the Cardedu farm. The *Prevotellaceae* family had a higher abundance in the Perdasdefogu and Villagrande farms (**Table 3**).

**Table 3:** Percentage of bacterial families divided by locality.

Percentage of bacterial families divided by locality																
	*Gracilibacteraceae*	*Erysipelotrichaceae*	*Clostridiales* Family XI. *Incertae* Sedis	*Christensenellaceae*	*Acidaminococcaceae*	*Peptostreptococcaceae*	*Clostridiaceae*	*Porphyromonadaceae*	*Prevotellaceae*	*Flavobacteriaceae*	*Eubacteriaceae*	*Ruminococcaceae*	*Lachnospiraceae*	*Rikenellaceae*	*Synergistaceae*	*Bacteroidaceae*
Baunei	41.05	27.47	30.70	42.26	31.31	33.13	33.95	27.98	27.39	23.84	27.57	32.78	31.92	29.87	39.26	29.28
Cardedu	31.19	25.27	19.01	19.31	112.07	5.44	25.63	60.69	32.78	60.00	33.14	23.28	20.04	23.83	22.86	35.26
Perdasdefogu	18.38	78.18	27.12	8.64	28.99	80.00	27.04	28.27	49.92	51.30	43.91	31.44	37.51	53.00	12.16	65.90
Talana	23.63	61.65	34.06	15.69	39.28	70.66	37.43	14.62	24.99	39.21	48.41	36.27	42.37	21.65	18.11	60.60
Urzulei	12.47	78.44	23.28	8.55	38.37	22.00	24.22	21.19	29.01	27.92	30.42	27.99	33.22	19.88	12.00	57.50
Villagrande	17.90	45.06	38.60	15.47	37.38	33.74	32.11	44.03	45.22	52.31	44.86	34.43	36.15	40.26	21.48	41.45
Total	426993.17	912717.22	193546.29	654987.33	188681.25	345908.74	3131878.20	396834.62	1243367.90	838260.23	1770284.92	2622805.10	1959362.02	436662.14	94912.87	4018615.04

The most represented genera were *Ruminococcus*, *Eubacterium*, *Roseburia*, and *Clostridium*. For the genera *Ruminococcus*, *Roseburia*, and *Clostridium*, a greater abundance was noted in the Talana farm and a lower abundance in the Cardedu farm. For the genus *Eubacterium*, no significant difference was noted in the various farms (**Table 4**).

**Table 4:** Percentage of bacterial genus divided by locality.

Percentage of bacterial genus divided by locality				
	*Ruminococcus*	*Eubacterium*	*Roseburia*	*Clostridium*
Baunei	20.82	14.14	15.95	13.61
Cardedu	18.61	11.13	9.38	9.97
Perdasdefogu	31.55	16.07	10.80	12.97
Talana	43.55	13.95	21.95	20.58
Urzulei	28.83	11.12	12.80	11.71
Villagrande	35.82	19.45	13.17	17.56

The most represented species were *Ruminococcus faecis*, *Ruminococcus gauvreauii*, *Eubacterium hallii*, *Roseburia faecis*, and *Clostridium lavalense*. For the species *R. faecis*, *R. gauvreauii*, and *C. lavalense*, there were a greater abundance in the Talana and Perdasdefogu farms and a lower abundance in the Cardedu farm. For the species *R. faecis*, there was a greater abundance in the Talana farm (**Table 5**).

**Table 5:** Percentage of bacterial species divided by locality.

Percentage of bacterial species divided by locality						
	*Ruminococcus*_sp.	*Ruminococcus_gauvreauii*	*Roseburia_faecis*	*Eubacterium_hallii*	*Clostridium_lavalense*	*Ruminococcus_faecis*
Baunei	9.65	13.94	18.64	18.01	13.74	15.65
Cardedu	9.37	7.08	14.59	19.02	3.26	8.69
Perdasdefogu	36.93	17.83	13.75	20.61	26.63	11.03
Talana	23.15	29.57	26.07	14.55	25.59	20.74
Urzulei	9.58	13.06	15.45	13.02	16.57	26.92
Villagrande	11.33	18.51	11.50	14.80	14.22	16.96

**Figure 3:**
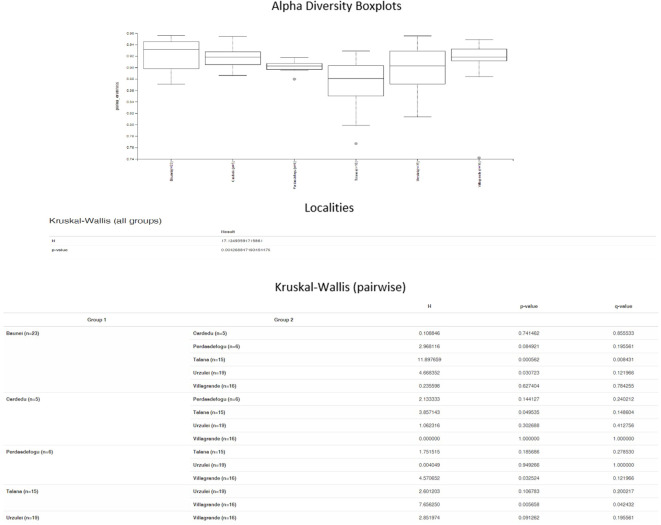
Alpha diversity boxplots.

Significant differences in the relative abundance of taxa were detected between the samples from Baunei and Talana (*P* = 0.0005) and Cardedu and Talana (*P* = 0.04) farms (**Figure 3**).

**Figure 4:**
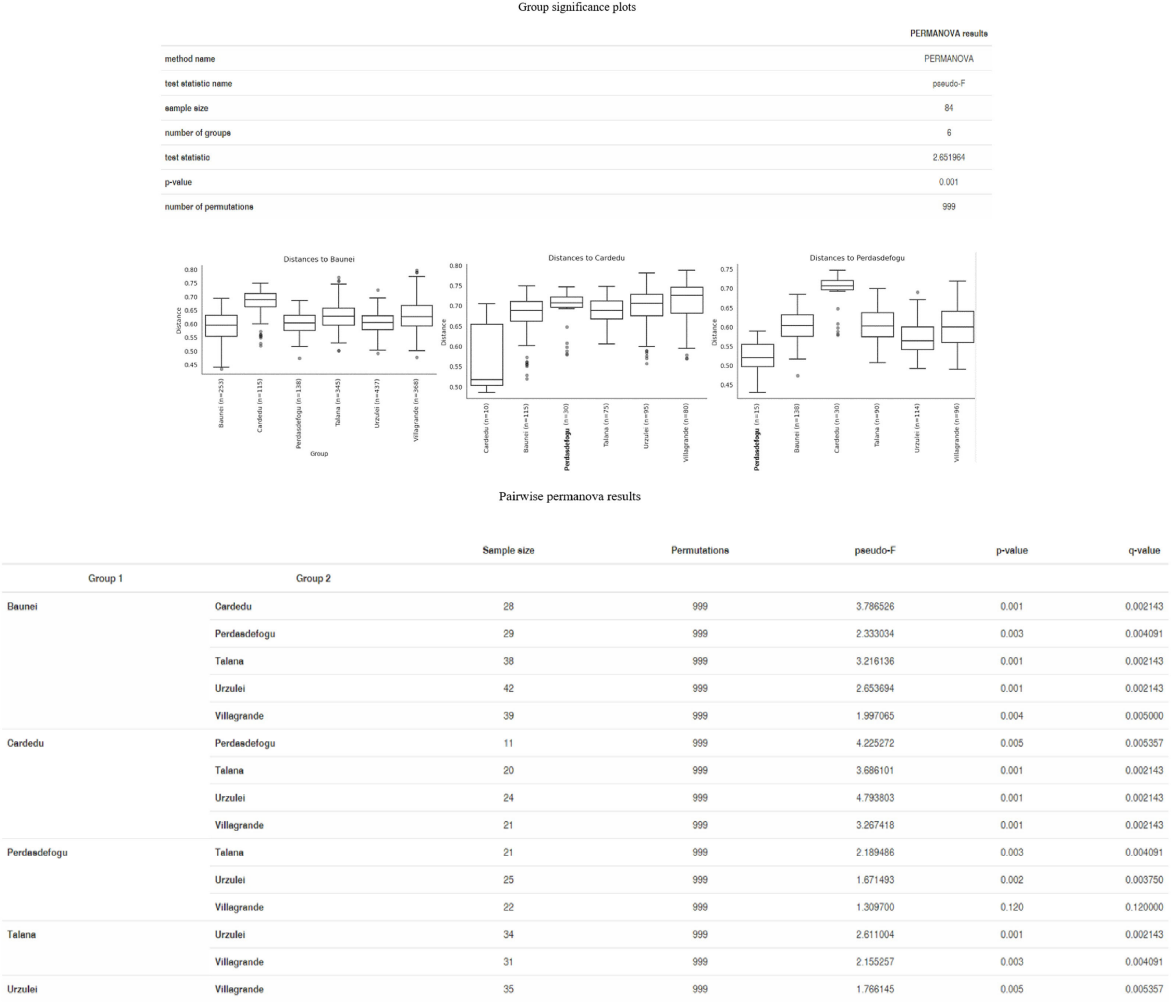
Group significance plots.

**Figure 5:**
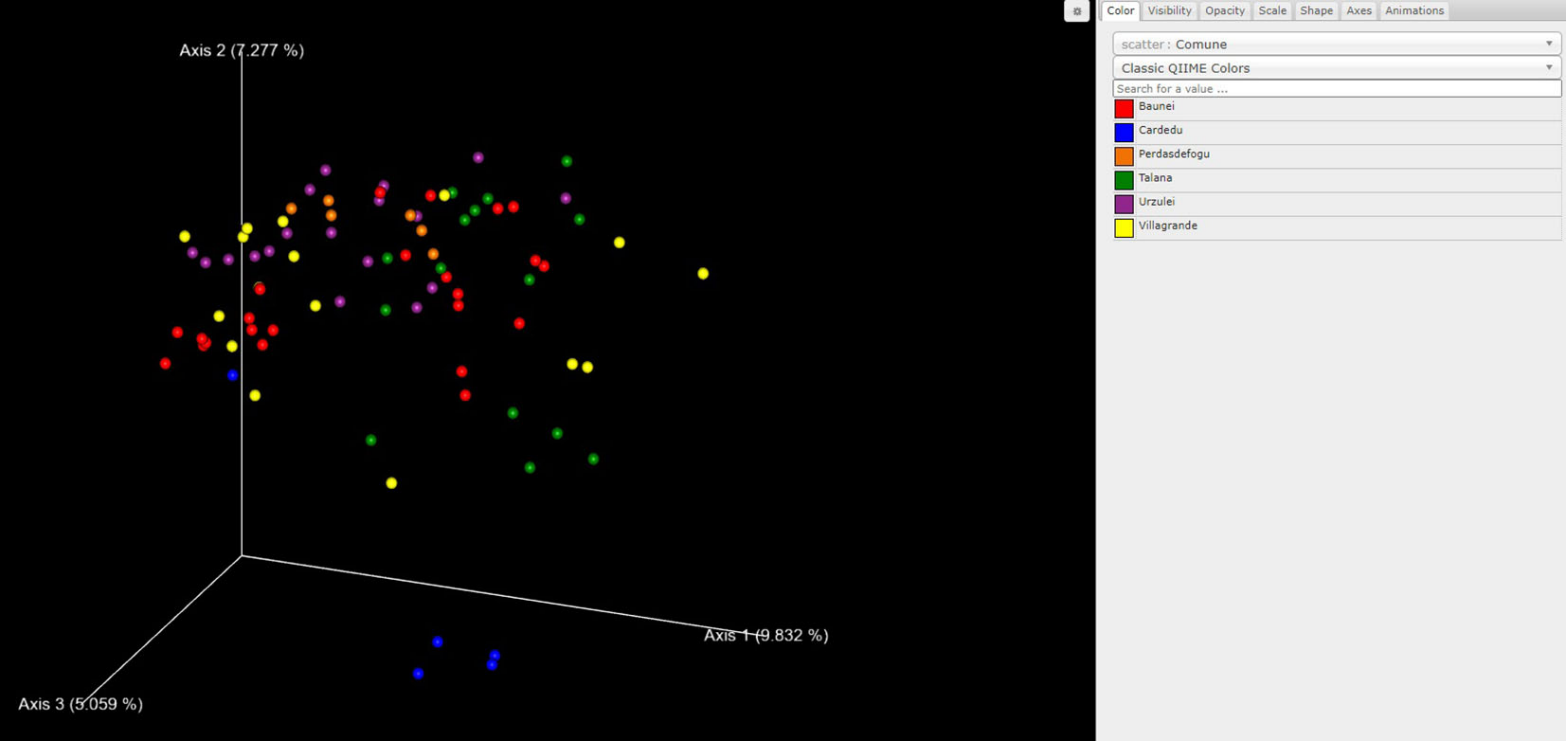
Beta diversity calculated with the weighted UniFrac metric to determine the distance between samples and PCoA to visualize the data.

**Figure 6:**
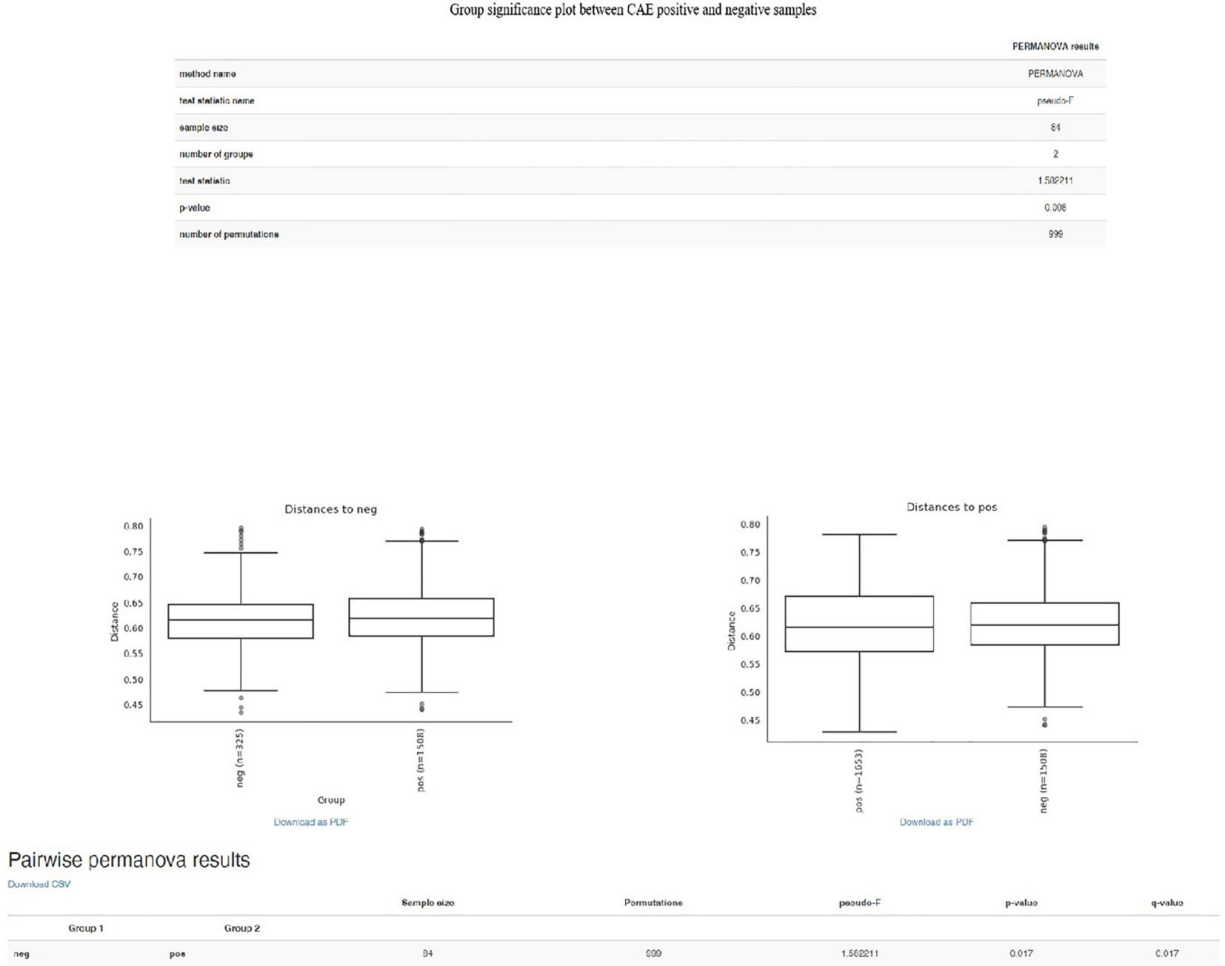
Group significance plot between CAE-positive and CAE-negative samples.

As far as beta diversity is concerned, the Cardedu farm was significantly different from the other sites, with *P*-values ranging from 0.001 to 0.005; similar results were obtained for the Baunei farm (**Figure 4**). A multidimensional sorting graph (**Figure 5**), where each sample is a point and the distance between the points represents the similarity, highlighted a cluster made up of the Cardedu locality. Beta diversity was significantly different between the CAE-positive and CAE-negative samples (*P* = 0.017) (**Figure 6**). On the farms in the localities of Perdasdefogu and Villagrande, *Prevotellaceae* were abundant; *Peptostreptococcaceae* and *Erysipelotrichaceae* were abundant in the localities of Perdasdefogu, Talana, Urzulei, and Villagrande. A significant abundance of *Acidaminococcaceae* on the Cardedu farm compared to that of the other families, a high abundance of *Porphyromonadaceae* and *Flavobacteriaceae*, and a decreased presence of *Peptostreptococcaceae* were detected (**Table 3**). In the samples from the locality of Talana, there was a prevalence of the *Roseburia* genus and *R. faecis* (**Tables 4**, **5**).

## 4 Discussion

*Prevotellaceae* was found in the localities of Perdasdefogu and Villagrande (Kim et al., 2014), where 4 out of 29 goats tested positive for CAE. From these data, we deduced that the presence of *Prevotellaceae* can be an indication of the disease, as highlighted by human clinical literature. The intestinal microbiome can exhibit bacterial hyperproliferation in rheumatoid arthritis (RA), which can be related to the onset or course of the disease. In particular, individuals affected by RA showed a high prevalence of *Prevotella* and *Prevotella* spp. The presence of these specific bacteria suggests a potential association between the intestinal microbiota and the development or exacerbation of this disease. Understanding the complex pattern of the relationship between the microbiome and RA could provide useful knowledge for the development of new therapeutic strategies targeting the intestinal flora, thus offering new perspectives for the management and treatment of this disease (Horta-Baas et al., 2017; Attur et al., 2022). Compared to FDR controls [first-degree relatives of RA patients (RA-FDR) have a higher risk of developing RA than the general population], individuals at risk of RA with systemic autoimmunity and/or RA-associated symptoms have an enrichment of *Prevotella* spp. The findings support the hypothesis of a mucosal origin in the development of RA. Intestinal dysbiosis could act as an early environmental modulator and may be a target of future preventive interventions (Alpizar-Rodriguez et al., 2019). These data may be related to the findings of studies on human individuals at risk of rheumatoid arthritis with systemic autoimmunity and/or symptoms associated with rheumatoid arthritis in which an increase in *Prevotella* spp. was found (Alpizar-Rodriguez et al., 2019). It could be hypothesized that the same scenario also occurs in goats affected by arthritic diseases, such as those analyzed.

Of interest is also the abundance of the genus *Roseburia* observed in the samples collected in the locality of Talana, higher than in other localities (**Supplementary Material 6**). Species belonging to the *Roseburia* genus are important inhabitants of the intestinal microbiome, and they are capable of fermenting complex polysaccharides into butyrate, a short-chain fatty acid that regulates the transepithelial transport of fluids, improves the oxidative and inflammatory state of the mucosa, influences human physiology, and serves as an energy source for colonocytes (Hillman et al., 2020).

**Figure 7:**
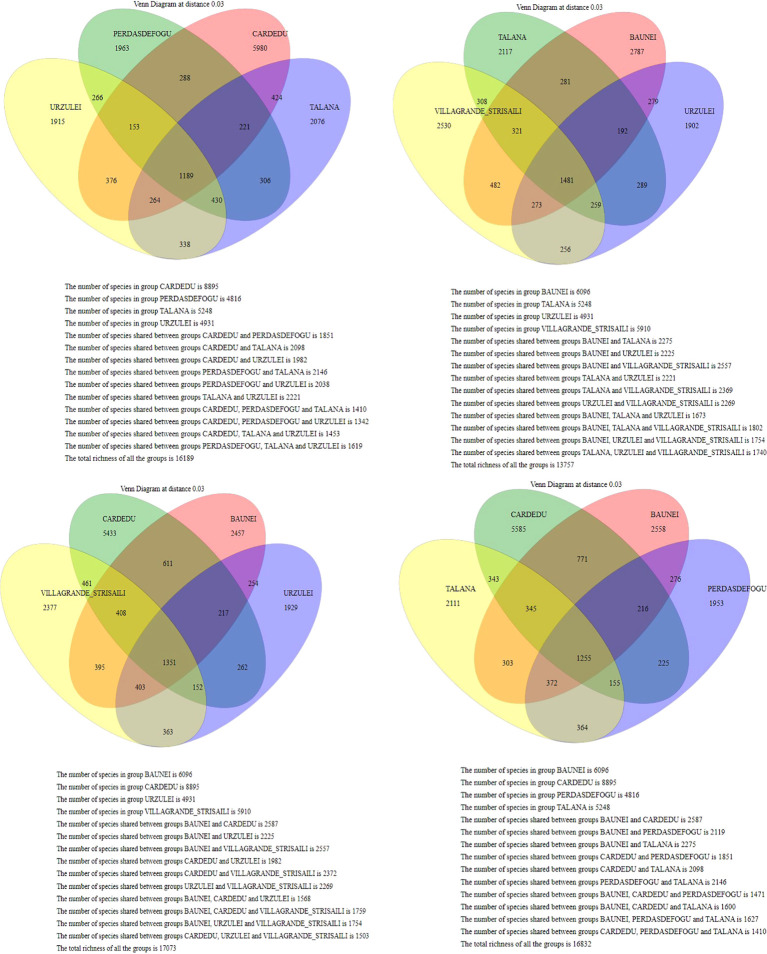
Venn diagram at a distance of 0.03; the Cardedu samples show a statistically significant difference compared to the other localities.

**Figure 8:**
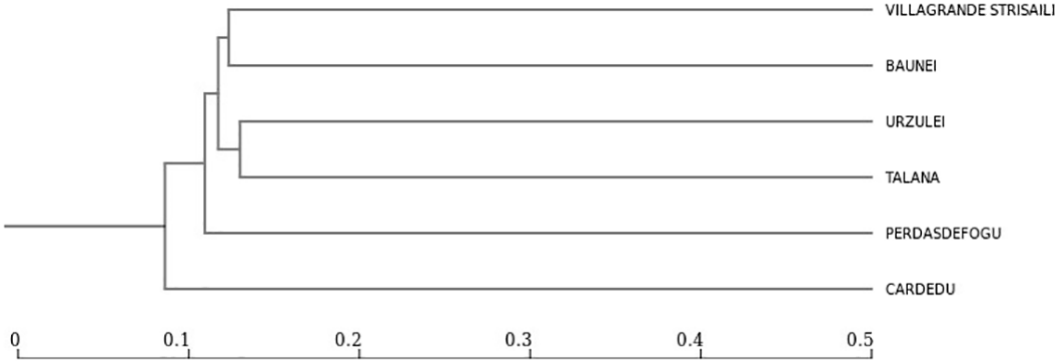
Dendrogram showing 4 clusters with greater distances between Cardedu and the other locality.

The only intensive farm located in Cardedu was statistically different from the other locations. In fact, out of a total of 8,895 identified species, only 3,310 were shared with other locations (**Figures 7**, **8**). In particular, the presence of *Acidoaminococcaceae* was significant (**Table 3**). This difference could be attributed to the farming system, the Cardedu farm being the only intensive one, and to the geographical distance of this location from the other sampling sites (**Figure 1**).

## 5 Conclusions

There are few studies in the literature regarding the fecal microbiome of goats and no studies with the same environmental and experimental conditions.

Therefore, the results of the present study suggest that extensive or intensive management of the farm can influence the intestinal microbiota of goats. The diversity of the farm of Talana could be attributed to the peculiarities of the rough and stony territory with many mines rich in copper, carbonate, and some veins of silver pyrite.

In the future, it would be interesting to compare the results obtained in the present work with those of other areas of Sardinia. To deepen the comparison between wild and intensive farming systems, further sampling would be necessary considering that in the present work, it was possible to analyze a limited number of samples for intensive farming.

It would also be desirable to monitor the goats from birth until a possible positivity for CAE to investigate the changes in the composition of the intestinal microbiome and the increase of *Prevotellaceae* to have new perspectives in the management of the disease.

## Data Availability

The original contributions presented in the study are publicly available. This data can be found here: Galaxy Server instance https://izs3.crs4.it/. To access the datasets, enter Email Address “guest@izs3.crs4.it” and Password “gU3st1ZS3CR$4”. Once logged in, click “Shared Data”. This will open a dropdown menu allowing access to Data Libraries and Histories.

## Supplementary material

Supplementary Material 1 Pipeline GALAXY.
