# Supplementary material for: Metagenomic analysis of goat feces from Ogliastra (Sardinia, Italy)
Source: Front Microbiomes. 2025 Jan 21;3:1474497. doi: 10.3389/frmbi.2024.1474497 (PMC12993504; doi:10.3389/frmbi.2024.1474497)
Supplement: Supplementary Material 1 — Pipeline GALAXY. [file DataSheet1.pdf]

Pipeline GALAXY

In our study we benefited from the ease of use and streamlined integration of Mothur version 1.39.5 within the Galaxy version 22.05 platform.

To perform our analysis, we adhered to the guidelines suggested by the authors of Mothur. Additionally, we followed an established pipeline “Chappid” that provides a structured framework for our analysis, incorporating best practices and methodologies for metagenomic investigations.

To start our analysis, we initially loaded the FASTQ files into collections based on their geographic origin in a new workspace within Galaxy.

A quality control was performed using FastQC (version 0.11.9). We used TrimGalore (version 0.6.7) [14] to remove low-quality reads and trim adapters from FASTQ files. Default parameters, including a quality threshold of 20 (QPhred).

To facilitate a multisample analysis we generated a group file in FASTA format using Mothur 's Make.group tool.

To simplify the computational processes we removed duplicate sequences using “ unique.seqs ” in Mothur.

Next, we used “ count.seqs ” to generate a table that determines the abundance of taxonomic classification and operational taxonomic units (OTUs) in subsequent analysis steps.

To evaluate the quality of the data, we used “ summary.seqs ” and observed that the majority of sequences fell within the range of 125 to 290 bases.

Next, we used the “ screen.seqs ” tool to systematically remove low-quality reads and sequences.

After aligning the cleaned sequences with the Silva reference database using Mothur 's “ align.seqs ” tool, we performed sequence analysis using the “ summary.seqs ” tool. At this point, we observed that most of the sequences were located between positions 6212 and 13871.

To ensure that all our readings overlapped exactly with the region of interest (positions 6212 to 13871), we conducted a series of data cleaning operations:

1. Screen.seqs: We used the “ screen.seqs ” tool to remove sequences that did not completely overlap the region of interest. This step is critical to ensure that only sequences relevant to our analysis are considered in subsequent steps.

2. Filter.seqs: Next, we applied the “ filter.seqs ” tool to eliminate any sequences that did not meet certain quality criteria. This helps maintain the consistency and quality of our dataset.

3. Unique.seqs: We used " unique.seqs " to remove redundancy from sequences, ensuring that each sequence is represented only once in our dataset. This step is important to further simplify the analysis and reduce computational complexity.

4. Pre.cluster: Finally, we performed pre-clustering using the “ pre.cluster ” tool. This step allows you to group nearly identical sequences into a single representation, further reducing noise in the data and improving the accuracy of subsequent analyses.

The entire data cleaning and preparation process is essential to ensure that subsequent analyzes are conducted on a reliable and representative dataset of our region of interest.

We used the command “ chimera.vsearch ” to identify and “ remove.seqs ” to remove chimeras from our data.

We proceeded to assign taxonomic classifications to the sequences. via “ Classify.seqs ” which uses RDP as the reference taxonomy.

We used " remove.lineage ". In this step, we manually selected specific taxonomic groups for filtering, which included chloroplasts, mitochondria, unknown, archaea, and eukaryotes.

We used “ Cluster.split ” with a taxonomic level setting of 4, corresponding to the Order level.

After clustering, we determined the number of sequences within each OTU of each group. using " Make.shared " with the limit of 0.03.

“ Classify.otu ”, provided us with a consensus taxonomy for each OTU.

To visualize our data we used Krona, which provides interactive visualizations of microbial community data. We performed the following activities:

We have converted the Mothur taxonomy file generated by the “ classify.otu ” tool into a Krona compatible format. with "Taxonomy -to- Krona ".

Krona pie charts : We ran the Krona wrapper for Galaxy developed at CRS4, specifically the " Krona pie chart tool ".

Krona plots per sample: To allow for a more detailed comparison of results between different samples, we have taken the additional step of creating corona plots per sample. To do this, we ran the “ classify.otu ” tool again with the “ persample ” option set to True. This option allowed us to find a consensus taxonomy for each sample group. Next, we ran the “Taxonomy-to- Krona” and “Crown Pie Chart” tools again for these per-sample classifications.

To ensure a fair comparison, we decided to normalize our dataset as some samples had more sequences than others:

Sequences counted per sample : Initially, we evaluated the number of sequences in each sample using the “ Count.groups ” tool, applied to the “shared” file generated in the previous step.

Subsampled for normalization: To normalize our data, we used the “ sub.sample ” tool. We set the subsampling size to match the size of the smallest sample in our dataset.

To estimate the alpha diversity of our samples, we first generated rarefaction curves. Rarefaction is a valuable measure that quantifies the number of OTUs observed as a function of subsampling size:

Calculation of rarefaction curves: Using the " Rarefaction.single " tool, we calculated the rarefaction curves. These curves were generated from the "shared" file created using the " make.shared " tool.

Rarefaction Curves: We visually represented the rarefaction curves using the "Plotting Tool – for multiple series and graph types" in the Galaxy environment. This graphical representation helps us visualize observed species richness across different subsampling sizes.

Summary Report – Finally, we used the " Summary.single " tool to generate a comprehensive summary report. This step involved randomly subsampling to the size of the smallest sequences (see previous step) and repeating this process 1000 times. The report included several key metrics:

sobs (https://www.mothur.org/wiki/Sobs) : observed richness (number of OTUs).

Coverage (https://mothur.org/wiki/Coverage) : Good coverage index (1 - (number of OTUs containing a single sequence / total number of sequences)).

Invsimpson (https://en.wikipedia.org/wiki/Diversity_index#Simpson_index) : inverse Simpson index (1/probability that two random individuals represent the same OTU).

Nseqs (https://www.mothur.org/wiki/Nseqs) : Total number of sequences.

These parameters provide valuable information about the diversity within our samples, including the number of unique OTUs, the completeness of our sampling, and the richness of the microbial communities.

Beta diversity is a critical measure that evaluates the similarity or dissimilarity in the membership and structure of microbial communities between different samples. In this section, we used two beta diversity calculators: thetaYC and the Jaccard index (jclass in Mothur) to capture different aspects of community similarity.

Here's a breakdown of the steps we followed:

Calculation of beta diversity: We calculated beta diversity using the “ Dist.shared ” tool. This tool rarefied our data, ensuring consistent subsampling for accurate comparisons. We used two beta diversity calculators: thetaYC and the Jaccard index (jclass in mothur) to evaluate community similarity.

ThetaYC (http://csyue.nccu.edu.tw/2005communicationindex.pdf)) measures similarity based on abundance data, while the Jaccard index (https://en.wikipedia.org/wiki/Jaccard_index)) considers presence-absence information.

Heatmap visualization with " Heatmap.sim ". The heat map allows us to observe patterns of similarity and dissimilarity between samples.Venn diagram: We generated a Venn diagram using the “ Venn ” tool to illustrate shared and unique OTUs across samples. This graphical representation helps us identify common and distinct microbial taxa across different sample groups.

Construction of the dendrogram: To further characterize the similarity of the samples, we constructed a dendrogram using the “ Tree.shared ” tool. This dendrogram was generated using both the jclass and thetaYC beta diversity calculators.
